# Multi-year time series of daily solute and isotope measurements from three Swiss pre-Alpine catchments

**DOI:** 10.1038/s41597-024-03192-5

**Published:** 2024-04-17

**Authors:** Julia L. A. Knapp, Tracy Napitupulu, Jana von Freyberg, Andrea Rücker, Bjørn Studer, Massimiliano Zappa, James W. Kirchner

**Affiliations:** 1 Department of Earth Sciences, Durham University, Durham, DH1 3LE UK; 2 Department of Environmental Systems Science, ETH Zurich, 8092 Zurich, Switzerland; 3 EPF Lausanne, Environmental Engineering Institute IIE, School of Architecture, Civil and Environmental Engineering ENAC, Lausanne, Switzerland; 4 Swiss Federal Institute for Forest, Snow and Landscape Research (WSL), Birmensdorf, Switzerland

**Keywords:** Hydrology, Environmental monitoring, Geochemistry

## Abstract

Time series analyses of solute concentrations in streamwater and precipitation are powerful tools for unraveling the interplay of hydrological and biogeochemical processes at the catchment scale. While such datasets are available for many sites around the world, they often lack the necessary temporal resolution or are limited in the number of solutes they encompass. Here we present a multi-year dataset encompassing daily records of major ions and a range of trace metals in both streamwater and precipitation in three catchments in the northern Swiss Pre-Alps. These time series capture the temporal variability observed in solute concentrations in response to storm events, snow melt, and dry summer conditions. This dataset additionally includes stable water isotope data as an extension of a publicly available isotope dataset collected concurrently at the same locations, and together these data can provide insights into a range of ecohydrological processes and enable a suite of analyses into hydrologic and biogeochemical catchment functioning.

## Background & Summary

Streamwater solute concentrations are strongly influenced by geology, land cover and land management as well as climate. Consequently, concentrations of dissolved substances such as inorganic compounds or nutrients in streamwater and precipitation are frequently used as indicators in catchment studies to understand flow paths and biogeochemical processes^1–3^. Investigating streamwater chemistry also allows understanding contamination events, because heterogeneity in flow paths and extended travel times mean that low-level contamination can be detected in streamwater for extended periods of time^4^. Streamwater solute concentrations can also exhibit slow changes and long-term trends due to variations in climate and land use^5,6^. Long-term records of streamwater chemistry can thus aid in understanding the relationships between land use impacts, climate, and water quality.

Long-term datasets of water chemistry have been recorded at numerous locations worldwide. These records include data from national monitoring programs, which are frequently publicly accessible, like the CAMELS-Chem dataset comprising U.S. Geological Survey water chemistry measurements^7^ or the Swiss National River Monitoring and Survey Program^8^. Hydrochemical data have also been collected regularly or episodically at experimental sites such as the Hubbard Brook Experimental Forest, US^9,10^, Plynlimon, UK^11,12^, or the Luquillo Critical Zone Observatory in Puerto Rico^13^. Water chemistry data are also available through large databases such as the GLObal RIver CHemistry database (GLORICH^14^), combining regular or episodic sampling from various locations worldwide.

The frequency of the data should typically be higher than that of the underlying processes to effectively capture and assess them. However, the collection, preparation, and analysis of water quality samples are costly and labor-intensive, and thus most long-term streamwater chemistry timeseries are only available at weekly or monthly intervals. High-frequency measurements at sub-hourly intervals using *in-situ* sensors, on the other hand, are often limited to a small number of chemical compounds and water quality parameters. These sensor-based datasets typically include temperature, dissolved oxygen, pH, conductivity, turbidity, nitrate and dissolved organic carbon^15^, and thus facilitate the investigation of only a limited range of catchment processes. Conversely, daily sampling can be considered a “sweet spot” between monthly or sub-hourly sampling frequencies. The daily time scale can capture the breadth of compounds across the periodic table and the temporal variability of flood events and snowmelt periods, while the manual labor required for sample collection, analysis, and processing remains manageable.

Here we present a dataset of streamwater and precipitation chemistry from three catchments in the northern Swiss Pre-Alps. Between June 2016 and May 2019, concentrations of major ions and a range of trace metals were measured daily in streamwater and precipitation in the Alp catchment and two of its tributaries, the Erlenbach and Vogelbach catchments. The time series was continued until May 2022 in the Erlenbach catchment. Each daily streamwater sample collected at the outlets of the three catchments is made up of four equal-volume samples collected at 6-hour intervals. While keeping manual labor low, this composite sample averages over the temporal variability of flow conditions better than a single daily grab sample does. Precipitation samples were collected as daily composite samples in the northern part of the Alp catchment near Einsiedeln from June 2016 to May 2019, and in the southern headwaters (Erlenbach) from June 2016 to May 2022. Daily discharge, air temperature, relative humidity, snow depths, and precipitation amounts are additionally provided in this dataset to facilitate its application, and complementary isotope analyses from the streamwater and precipitation samples, previously published by von Freyberg *et al*.^16^, have also been included. Long-term hydrological records are additionally available for these sites, dating back as far as the late 1960s in some cases^17^.

## Methods

### Site description

**Fig. 1:**
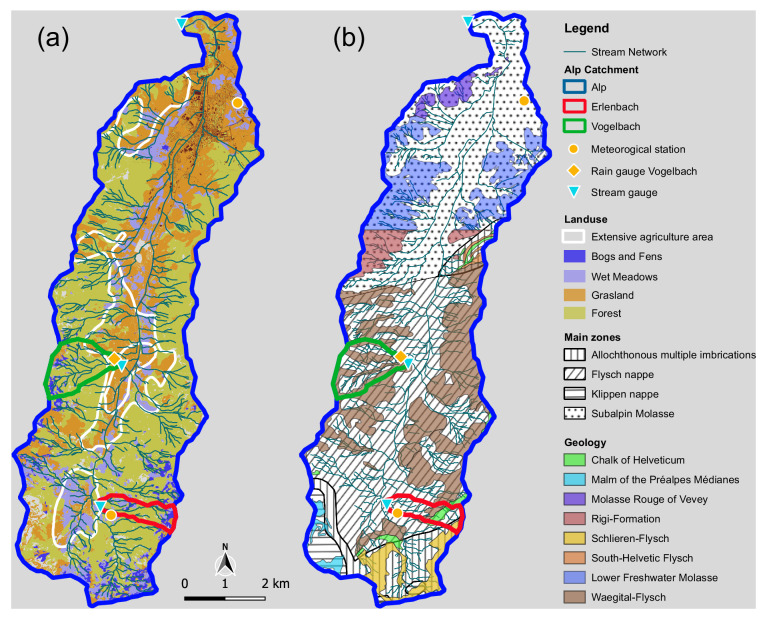
Site map. (**a**) Landuse as derived from Price *et al*.^29^ and (**b**) Geology (based on GeoMaps 500; Federal Office of Topography swisstopo, Bern, https://www.swisstopo.admin.ch/en/geomaps-500-vector).

Stream water samples were collected from the discharge stations at outlets of the Alp, Vogelbach, and Erlenbach catchments. The 46.4 km^2^ Alp catchment ranges in elevation from 840 to 1898 m above sea level. It is located approximately 40 km south of Zurich and is bounded to the south by the Mythen Mountains as its highest point. The Vogelbach and Erlenbach are sub-catchments of the larger Alp catchment. The Vogelbach is situated on its western side, with an area of 1.6 km^2^ and an elevation ranging from 940 to 1480 m above sea level; the Erlenbach is located on the eastern side of the Alp catchment and covers an area of 0.72 km2 with an elevation between 1080 and 1520 m above sea level. Two meteorological stations are located inside the Alp catchment, one near the Alp outlet (at 910 m a.s.l) and a second one in the Erlenbach catchment (at 1228 m a.s.l.). An additional rain gauge is located inside the Vogelbach catchment (at 1145 m a.s.l.). The locations of the catchments and all river and rain gauging stations are shown in Fig. 1.

The study area has a temperate climate, characterized by a distinctive increase of precipitation with altitude. The mean annual rainfall between 1969 and 2019 was 2266 mm/year in the headwater catchments^17^ whereof one-third fell as snow^18^, and 1723 mm/year in the flat region of the northern part of the Alp catchment at the meteorological station near the Alp outlet^17^. The average monthly air temperature varied from −1.9 °C in February to 15.9 °C in July at the Erlenbach meteorological station during those years, and was slightly higher at the Einsiedeln meteorological station for the same months (with average monthly temperatures of −1.2 °C and 17.7 °C in February and July, respectively).

The catchments are steep (20–40°) in particular in the headwaters^17^, and land cover is dominated by spruce and fir forests, wet meadows, as well as grasslands. Some urban and agricultural areas are present, in particular in the lower areas of the Alp catchment^17,19^ (Fig. 1a). The soils in the headwaters are mostly used for cattle and sheep grazing, whereas the lower areas of the Alp catchment are suitable for crop cultivation, which may result in the application of fertilizers and manure. Road salt is used in the winter, but no paved roads are present in the headwater catchments. The bedrock is predominantly made up of three different types of Flysch (Waegitaler Flysch, Wild Flysch and Schlieren Flysch), consisting of alternating layers of conglomerate and calcareous sandstones with schists and marlstones^20^, with some local differences in bedrock between the catchments. Further details on the geology and bedrock distribution are illustrated in Fig. 1b. Soils consist of low-permeability mollic gleysols in flatter areas of the catchment where the water table is close to the surface^21^, and umbric gleysols on the steeper slopes and ridges^22,23^.

### Precipitation and streamwater sampling

**Fig. 2:**
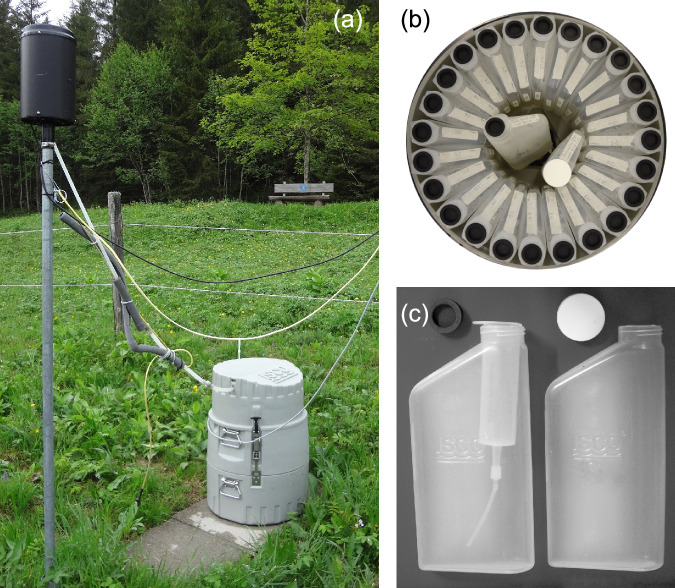
Sampling setup. (**a**) Daily sampling at the Erlenbach meteo station with rain gauge placed above an ISCO Sampler, (**b**) ISCO Sampler with 24 ISCO bottles and two control bottles in the middle and (**c**) ISCO bottle with (left) and without (right) evaporation protection modification. (**c**) taken from von Freyberg *et al*.^24^ with permission.

Streamwater samples were collected from June 2016 to May 2019 at the outlets of the Alp and Vogelbach catchments and between June 2016 and May 2022 at the Erlenbach outlet. Samples were collected into clean 1-litre HDPE bottles (Fig. 2b) using an automatic water sampler (6712-Fullsize Portable Sampler, Teledyne ISCO, Lincoln, Nebraska, USA; Fig. 2a). Each HDPE bottle was equipped with an evaporation protection system^24^ to avoid sample loss, evapo-concentration, and isotopic fractionation. Each daily streamwater sample consisted of four 100ml-samples that were pumped from the stream every 6 hours starting at 5:40 a.m. The filled sample bottles were retrieved from the field sites every 21 to 24 days and brought to the laboratory at the Department of Environmental System Science of ETH Zurich for chemical analysis.

Precipitation samples were collected between June 2016 and May 2019 at the meteorological stations in Einsiedeln and between June 2016 and May 2022 at Erlenbach as cumulative integrated samples from 5:40 a.m. until 5:39 a.m. the next day. Rainfall samples were collected into an automatic water sampler (6712-Fullsize Portable Sampler, Teledyne Isco, Lincoln, Nebraska, USA; Fig. 2a) via a silicon tube, through which water drained into the autosampler by gravity. HDPE sampling bottles were equipped with the same evaporation protection system as was used for streamwater sampling. During the snow-free season of 2016 and 2017, rain was captured with a plastic funnel with a 13.5 cm diameter at both sites. During the 2016/2017 winter season, the plastic funnels were replaced with 30-cm long, 15 cm diameter extended aluminum funnels with a heating cable wrapped around the silicon tube to avoid any freezing of the rain samples or freezing and sublimation of the accumulated snow. At the beginning of September 2017, heated rain gauges (52202 Electrically Heated Rain and Snow Gauge, Campbell Scientific, Loughborough, UK), that simultaneously served as precipitation collectors, replaced the funnels at both stations. The rain collectors were additionally equipped with a factory-supplied heating pad under the tipping bucket due to insufficient heating of the tipping bucket mechanism in very cold periods. The heating pad was activated when the air temperature fell below 4 °C. Moreover, the housings of the collectors were painted black to support the melting process when exposed to solar radiation. More detailed information on both the streamwater and precipitation sampling procedure has been published in von Freyberg *et al*.^16^.

### Sample handling and chemical analysis

All stream- and rainwater samples were stored in sealed autosampler ISCO bottles and refrigerated at 4 °C in the laboratory until preparation for analysis. Before the chemical analysis, the samples were passed through 0.45-µm Teflon filters (DigiFilter micron Teflon, S-Prep GmbH, Ueberlingen, Germany or WIC 80345, WICOM, Heppenheim, WICOM Germany GmbH). The filtrates were divided into three aliquots for chemical analyses. For the anion measurements, samples were filled into 1.5 ml autosampler glass vials (VWRRI548-003, VWR International, Dietikon, Switzerland) with silicon seals (11 mm Alu-Crimp Caps WIC 44200/100, WICOM International AG, Maienfeld, Switzerland). For the analysis of cations, about 10 ml of the filtered sample was acidified using 50 µl of 1% ʋ/ʋ concentrated nitric acid in a 15 ml sterile plastic tube (7.188.271 Centrifuge tubes, Polypropylene, Cellstar, Greiner Bio-one International GmbH, Kremsmuenster, Austria). The nitric acid had been purified by redistillation. The third aliquot was used for stable water isotope analysis (the analyses of the isotope data are described elsewhere, please refer to von Freyberg *et al*.^16^, for details). The samples were analyzed in batches, typically consisting of 3 weeks of samples from all five sites collected from the field on the same day, i.e., up to 5 × 24 = 120 samples, with the exact number depending on the pick-up day and the number of days with precipitation.

Major anions (chloride, nitrate, sulfate) were analyzed with ion chromatography (940 Professional IC Vario, Metrohm AG, Herisau, Switzerland, instrument precision approx. 0.1–1 ppm) and a conductivity suppressed anion self-regenerating suppressor using 100 mM sulfuric acid as regenerant. In each injection, 200 µL of the sample was eluted for 18 min with anhydrous 3.2 mM sodium carbonate and 1.0 mM sodium bicarbonate as eluent (Fluka, 71350 and 71628, respectively) and an isocratic flow rate of 0.7 mL/min using Metrosep A Supp 5 as the separation column (Metrohm 250/4.0, 6.1006.530). Column temperature was maintained at 30 °C. Prior to the sample analysis of each batch, a five-point calibration curve of each anion was constructed using Multi Anion Standard 1 (Sigma Aldrich, 69734, 100 mL) with dilutions in nanopure water: 0.250 to 10 mg/L for chloride, and 0.500 to 20 mg/L for nitrate and for sulfate. The concentration of each anion was determined from the slope of the linear regression through the origin which was fitted to each calibration curve. For additional validation, a quality control standard (commercial standard natural water 160150; “Volvic”) was measured at irregular intervals throughout the analysis. Each area under the injection peak in the chromatograms was integrated manually using the software IC Net (Methrohm AG, Herisau, Switzerland). Detection limits for all anions were 0.1 mg/L. Please note that any samples collected before 25^th^ Jan 2017 (or 6^th^ March 2017 at Alp Outlet) were not analysed for anions.

Major cations (sodium, potassium, magnesium, and calcium) and trace metals (barium, chromium, copper, iron, and strontium) were measured with an Agilent 7900 quadrupole Inductively Coupled Plasma-Mass Spectrometer (ICP-MS, Agilent Technologies, Santa Clara, CA, USA) equipped with a micro concentric nebulizer (Glass expansion, A13-1-UM04X, 0.4 mL/min flow rate). The measurements were performed with the included Octopole Reaction System (ORS) in either helium collision mode or hydrogen reaction mode except for Barium and Calcium (Table 1). The linear calibration curve consisted of seven points using ICP multi-element standard solution X (Supelco, 100 mL, 1094930100). After every 20 samples, a quality control standard (Multi-Element standard solution for ICP-MS, CPAchem, 100 mg/L, Ref-No. MB56A.K1.5 N.L1) was used to validate the calibration. Measurement uncertainties are provided in Table 1.

**Table 1:** Measurement uncertainties associated with the measured cations.

Determinand	Chemical symbol	Detection limit [µg/L]	ICP-MS mode
Barium	Ba	1	No Gas
Calcium	Ca	1	No Gas
Chromium	Cr	0.50	He^*^/H_2_^⁑^
Copper	Cu	1	He^*^/H_2_^⁑^
Iron	Fe	0.1	H_2_
Potassium	K	0.1	He^*^/H_2_^⁑^
Magnesium	Mg	0.1	He^*^/H_2_^⁑^
Sodium	Na	0.1	He^*^/H_2_^⁑^
Strontium	Sr	0.005	He^*^/H_2_^⁑^

^*^until 19 Jan 2021.

^⁑^after 19 Jan 2021

Detection limits are calculated as 10 times the standard deviation of 10 injections of the blank (nanopure).

Outliers were removed based on expert judgment. Exclusions were done either for individual extreme outliers, or batch-wise (i.e., indicating that something went wrong during sample collection or preparation, or during the analysis of a whole batch) if outliers were observed for more than one element. High relative standard deviations of ICP-MS measurements were also considered as indicators of potential outliers and the measurement was removed if necessary. Care was taken to only remove outliers from hydrologically stable periods as to not remove true variability arising from e.g. storm event responses. Removed outliers were replaced by −9999 in the dataset.

### Hydrometeorological data

The dataset includes daily averages of river discharge (calculated between 5:40 am and 5:39 am of the next day) at the Erlenbach and Vogelbach catchment outlets (Fig. 1). These were calculated from 10-min measurements provided by the Swiss Federal Institute for Forest, Snow and Landscape Research (WSL). The design of the stream gauge at the Vogelbach does not allow for reliable stream discharge measurements at low flows, and thus unrealistically low discharge data at this station have been interpolated using measurements from the Alp River. Discharge data from the Alp River at the city of Einsiedeln, which was provided by the Swiss Federal Office of the Environment (FOEN) at 10-min resolution, was aggregated to daily averages.

Daily precipitation amounts were recorded with a heated rain gauge at the Erlenbach meteorological station (Fig. 1). Precipitation measurements cannot distinguish between rain and snow, as the rain gauge is heated during the cold seasons. In the dataset we also provide snow depth (recorded daily at 6 am) and daily averages of air temperature and relative humidity measured at the Erlenbach meteorological station by WSL. Daily precipitation amounts in the lower Alp catchment were obtained from a meteorological station near the city of Einsiedeln. This station is operated by the Swiss Federal Office of Meteorology and Climatology (MeteoSwiss).

All hydrometric and meteorological data provided with the dataset cover the period of the solute records (i.e. from June 2016 to May 2019 in the Vogelbach catchment and from June 2016 to Mai 2022 in the Erlenbach catchment). Due to legal restrictions, however, we cannot provide any of the data from FOEN or MeteoSwiss (i.e., Alp River discharge and precipitation fluxes at Einsiedeln). These, however, can be requested free of charge for research and educational purposes from FOEN (https://www.bafu.admin.ch/bafu/en/home/topics/water/state/data/obtaining-monitoring-data-on-the-topic-of-water/hydrological-data-service-for-watercourses-and-lakes.html) and MeteoSwiss (https://gate.meteoswiss.ch/idaweb/login.do).

The dataset also includes daily, basin-averaged precipitation amounts estimated from the rainfall-runoff model PREVAH using the WINMET pre-processing tool^25^, based on data from more than 30 rain gauges in the area. The calculation details can be found in von Freyberg *et al*.^16^.

## Data Records

The data are publicly available from figshare^26^ as a text file. The dataset is a table with 7557 rows and 39 columns with concentrations of major ions (sodium, potassium, magnesium, calcium, chloride, nitrate and sulfate) and trace metals (barium, chromium, copper, iron and strontium) for each sample. In total, the file contains 51816 hydrochemistry data with 759 outliers denoted as –9999. Additionally, stable water isotope measurements in streamwater and precipitation are included in the dataset. Daily time series of key hydrologic and meteorologic variables are also included, such as daily streamwater and precipitation fluxes, air temperature, relative humidity and snow depth. The columns of the dataset are explained in Supplementary Table 1, providing information on the sampling time, location, and water source of each sample. The isotope timeseries are described in detail by von Freyberg *et al*.^16^.

## Technical Validation

### Solute concentrations in streamwater and precipitation

**Fig. 3:**
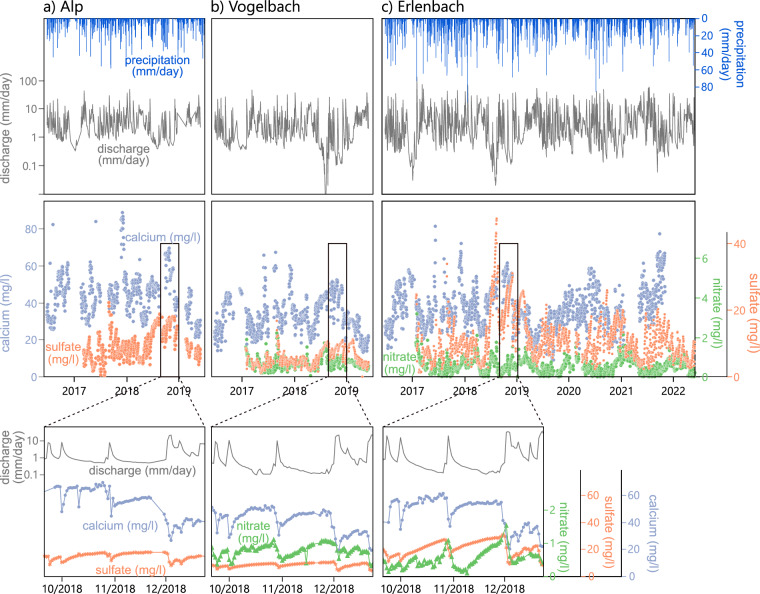
Water fluxes (top) and streamwater concentrations of calcium, sulfate and nitrate (center), and four-month detailed views of discharge, calcium, sulfate, and nitrate concentrations (bottom) at the Alp (**a**), Vogelbach (**b**), and Erlenbach (**c**). No nitrate data is plotted for the Alp River due to vastly different concentration ranges overall (see Fig. 4b).

**Fig. 4:**
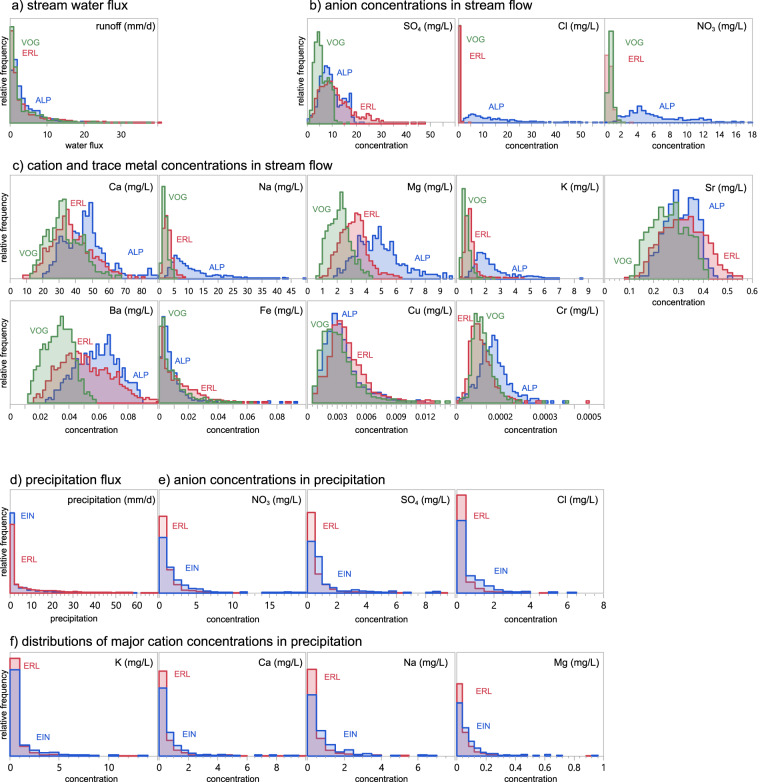
Solute concentrations in streamwater and precipitation. Distributions of water fluxes (**a**), and anion (**b**) as well as cation and trace metal (**c**) concentrations in streamflow at the outlet of the Erlenbach (red), Vogelbach (green) and Alp (blue) catchments, and precipitation fluxes (**d**), anion concentrations (**e**), and cation concentrations (**f**) in precipitation at the Erlenbach (red) and Einsiedeln (blue) meteo stations. Distributions show the relative frequency of different concentrations. Solutes in (**b**) and (**c**) are ordered by decreasing mean concentration across all three sites. Please note that some outlier concentrations are not included in the distributions for better visualization. Y-axes are scaled individually for each plot.

Streamwater solute concentrations at all three sites are highly variable over time (Fig. 3), illustrating that the dataset captures a range of discharge and wetness conditions. Average streamwater concentrations and their distributions differ among catchments (Fig. 4a–c), reflecting site differences in land use, geology, and human influence and consequentially in the application of fertilizer and road salt (see Site Description). Solute concentrations in precipitation are generally lower, but typically more variable over time, than those in streamwater. At the same time, spatial variability in precipitation concentrations is low, as illustrated by the rather similar concentration distributions in precipitation at Erlenbach and Einsiedeln (Fig. 4d–f).

### Evaluation of storage effects

**Fig. 5:**
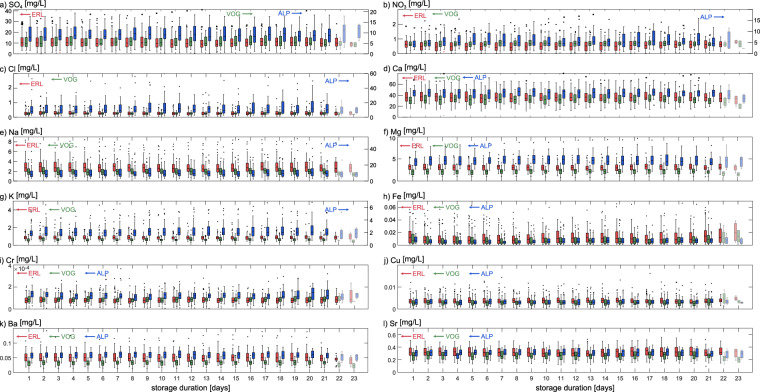
Impact of storage duration on solute concentrations. Upper and lower edges of each box represent the 25^th^ and 75^th^ percentiles of the concentrations, respectively, and the central mark indicates the median. The whiskers extend to the most extreme data points that are not considered outliers, and the outliers are plotted individually (any datapoint that extends outside the 25^th^ or 75^th^ percentile by more than 1.5 times the interquartile range is considered an outlier here). Each box is normally based on more than 20 individual samples (average sample number per storage duration is *n* = 75.4 at Erlenbach, *n* = 35.4 at Vogelbach and *n* = 26.5 at Alp); only boxes shown in lighter colors at very long storage durations of 22 and 23 days are based on less than 10 samples. For the concentration axes assigned to each sampling site, please refer to the small arrows inside the subplots.

All samples were stored for extended periods of time (up to 24 days) inside the ISCO autosamplers in the field without cooling prior to retrieval. Although the ISCO sample bottles were protected against evaporation^24^, chemical reactions could potentially have occurred during this time which may have impacted the sample concentration, in particular for nutrients such as nitrate. To evaluate a possible storage effect, we calculated sample concentrations as functions of storage duration across the whole duration of the time series. The results of this analysis are illustrated in Fig. 5. While sample concentrations vary naturally in the different streamwater samples resulting in large ranges of concentrations overall, we observe no distinct trends with storage duration. The apparent decrease in concentrations at very long storage durations (e.g., for calcium at 22 and 23 days of storage) is likely an artefact resulting from the very small sample numbers used to calculate the average concentration at this storage duration. Hence, while sample storage of up to 21 days in the field cannot be considered ideal, it does not appear to impact streamwater concentrations in our data.

### Comparison to high-frequency and weekly data

To validate our streamwater chemistry data, we compared them with two other datasets collected at the same locations but at different temporal resolutions. The National River Monitoring and Survey Program (NADUF) of the Swiss Federal Office for the Environment (FOEN) tracks a range of water quality parameters in Swiss rivers, in some instances since 1972. NADUF samples are collected as weekly flow-weighted composite samples at the gauging stations of the Erlenbach and Vogelbach catchments and analyzed for major ions (but not trace metals). All NADUF water samples are analyzed at the laboratory of the Swiss Federal Institute of Aquatic Science and Technology (Eawag). Additionally, high-frequency (approximately every 30–60 min) *in-situ* isotopic and chemical analyses of precipitation and streamwater have been taking place at the Erlenbach catchment outlet since early 2017^27,28^.

**Fig. 6:**
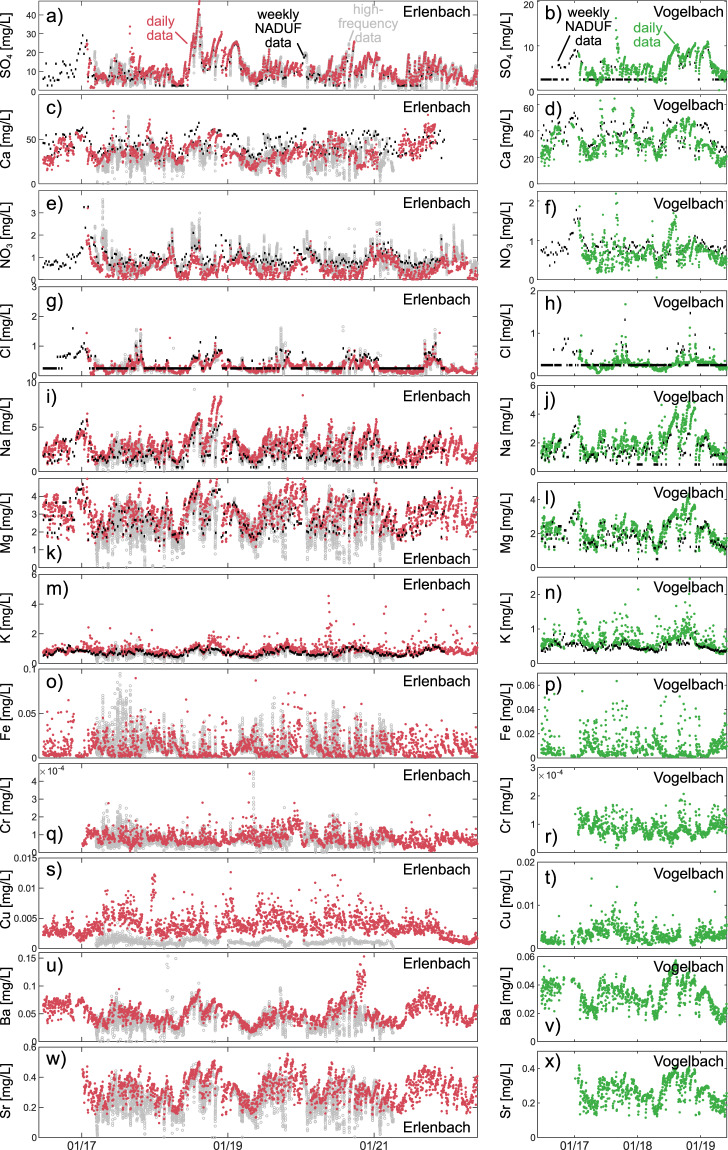
Comparison of timeseries of daily streamwater chemistry data from Erlenbach (red) and Vogelbach (green) with the corresponding 7-day volume-weighted NADUF concentrations (black) and those obtained from high-frequency sampling (grey, Erlenbach only).

Although we expect an approximate agreement of our daily streamwater chemistry data with both the weekly NADUF and the high-frequency data, some discrepancies are likely due to methodological differences in the data collection and sample analysis. Our daily streamwater samples are composited from four equal-volume daily subsamples. Conversely, each weekly NADUF sample is composited from approximately 30–750 individual subsamples collected flow-proportionally into one refrigerated bottle, whereas the high-frequency dataset is made up of instantaneous streamwater grab samples. Further discrepancies may arise due to differences in the storage and analysis procedures, so a perfect match between daily, weekly, and high-frequency data cannot be expected. Nonetheless, Fig. 6 demonstrates a general agreement between the temporal dynamics in concentrations across all three datasets. Moreover, our daily samples yield measurable concentrations when the NADUF dataset defaults to the detection limit, as is the case in the NADUF data for 13% and 73% of the sulfate samples at Erlenbach and Vogelbach, respectively, during the time span of our dataset, as well as 64% and 56% of the chloride samples at Erlenbach and Vogelbach, respectively.

The time series in Fig. 6 illustrate the general coherence between the daily, weekly and high-frequency data, which can be seen as indicating the reliability of our daily dataset. This daily dataset also includes trace elements (strontium, barium, iron, copper, and chromium) that are absent from the NADUF dataset.

### Supplementary information

Supplementary Table 1

## Data Availability

No custom code was used to process the data described in this paper.
